# Jump–Decline Evolution of Transcription Factor-Type Master Sex-Determining Genes

**DOI:** 10.3390/genes17101305

**Published:** 2026-10-20

**Authors:** Michihiko Ito, Kei Nakasako

**Affiliations:** Department of Bioscience, School of Science, Kitasato University, Sagamihara 2520373, Japan

**Keywords:** gene duplication, turnover, threshold, transcription factors, sex-determining genes, nearly neutral evolution, genetic takeover

## Abstract

Master sex-determining genes (MSDGs) occupy the top of developmental hierarchies and act as molecular switches that initiate female or male differentiation. Despite their essential roles, MSDGs exhibit rapid evolutionary turnover across vertebrates. In this review, we examine the molecular evolutionary trajectories of three independently evolved vertebrate MSDGs—*Sry* in mammals, *dmy* in medaka fishes, and *dm-W* in *Xenopus* frogs—and assess the extent to which their evolutionary histories are consistent with initial functional innovation followed by relaxed functional constraint, possible tolerated functional decline, and potential replacement or loss. Regulatory rewiring, structural innovation, or key amino acid substitutions can generate a functional “Jump” that enables a newly arisen gene to cross the threshold required for sex determination and become established as a master regulator. Once the new sex-determining system is established, further increases in MSDG activity may provide little additional fitness advantage, allowing purifying selection to relax. Nearly neutral or mildly deleterious substitutions can then accumulate while residual activity remains sufficient for normal sex determination, producing a tolerated functional “Decline.” Importantly, replacement need not await a decline in ancestral MSDG activity below the functional threshold. A newly emerging MSDG may become established through competition within a population while the ancestral determinant still retains sufficient sex-determining activity, thereby rendering the ancestral MSDG dispensable and permitting its subsequent functional decay or loss. Together, these cases suggest that the switch-like, threshold-dependent architecture of sex determination can permit initial innovation followed by relaxed functional constraint and, in some lineages, turnover of upstream master regulators. We propose “Jump-Decline” evolution as a testable conceptual framework linking the birth, functional relaxation, and potential turnover of vertebrate MSDGs.

## 1. Introduction

Gene duplication is a major mechanism generating evolutionary novelty. Since Ohno’s classical proposal [1], duplication has been recognized as a process that can relax purifying selection on one gene copy, thereby permitting functional diversification. Over the past half century, neofunctionalization, subfunctionalization, and pseudogenization have been widely recognized as three major evolutionary fates of duplicated genes [1,2,3,4,5,6]. These frameworks are particularly relevant to the evolution of master sex-determining genes (MSDGs), many of which have arisen through duplication, translocation, or regulatory modification of pre-existing developmental regulators.

In the neofunctionalization model, one duplicate acquires a novel regulatory or biochemical function while the other retains the ancestral role. This framework provides a natural explanation for the origin of several MSDGs, in which a duplicated or modified developmental regulator acquires a new position at the top of the sex-determining hierarchy. Subfunctionalization, in contrast, describes the partitioning of ancestral functions between duplicates [3], whereas pseudogenization involves the loss of gene function through the accumulation of disabling mutations, often following relaxation of selective constraint [7]. Although these frameworks explain major outcomes of duplicate-gene evolution, they do not, by themselves, explicitly describe the temporal sequence emphasized here: a newly arisen MSDG acquires enhanced or novel sex-determining activity and becomes established, then may subsequently undergo tolerated functional decline, and may ultimately be replaced by another determinant or lost. This perspective connects functional innovation and degeneration as successive phases that can occur within the evolutionary history of a single MSDG lineage.

Despite these advances, a fundamental question remains unresolved: why can MSDGs evolve so rapidly and tolerate substantial molecular change despite their essential role in determining sexual fate? Many genes involved in fundamental cellular and developmental processes are subject to strong purifying selection because reductions in molecular activity can directly compromise organismal fitness. Yet several MSDGs exhibit rapid sequence evolution, lineage-specific functional divergence, and recurrent loss or replacement despite occupying the top of the sex-determining hierarchy [8]. This apparent paradox may reflect the distinctive architecture of sex-determining systems. MSDGs often function as developmental switches: once their activity exceeds a functional threshold during a critical developmental window, the downstream female- or male-differentiation pathway can be initiated [9]. Selection may therefore act primarily to maintain MSDG activity above this threshold rather than to maximize molecular performance. Under such conditions, activity-reducing mutations could accumulate without immediately compromising developmental output, allowing substantial molecular evolution while sex-determining function is retained.

Our previous studies on vertebrate MSDGs provide empirical support for this possibility. In *Oryzias* fishes and *Xenopus* frogs, functional analyses of *dmy* and *dm-W* revealed that specific amino acid substitutions can substantially alter the molecular activity of these newly evolved sex-determining factors [10]. More recently, comparative and functional analyses of mammalian *Sry* demonstrated pronounced relaxation of purifying selection and lineage-specific reductions in molecular activity, supporting a process we termed “tolerated functional decline” [9]. Together, these findings raise the possibility that independently evolved vertebrate MSDGs may share aspects of a common evolutionary trajectory: functional or regulatory innovation during their establishment, followed in some cases by relaxation of selective constraint and possible functional decline.

**Figure 1:**
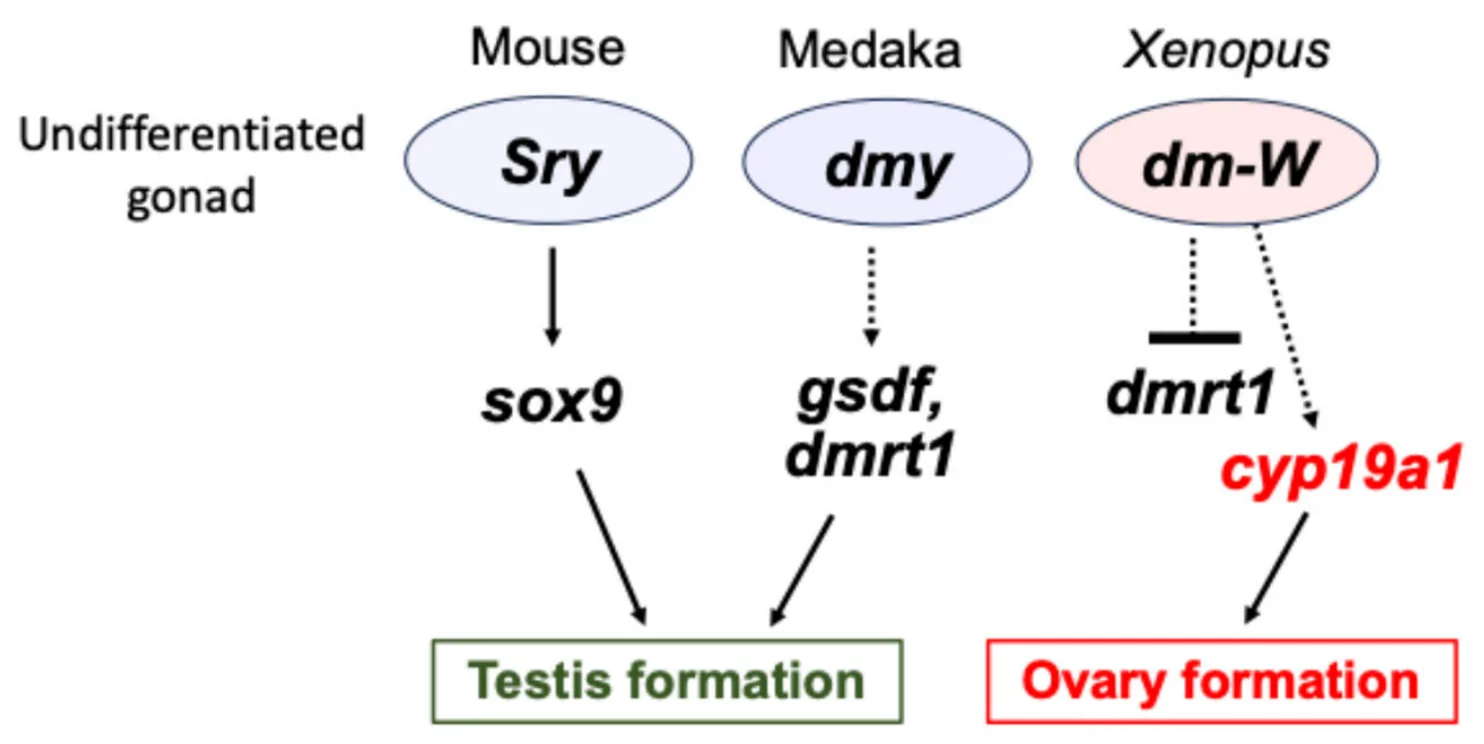
Comparison of developmental regulatory systems controlled by *Sry*, *dmy*, and *dm-W*. Schematic comparison of the gonadal sex-determining pathways in mice, medaka, and *Xenopus*. In mice, SRY directly activates *Sox9*, promoting testis formation. In medaka, DMY acts upstream of male-promoting factors, including *gsdf* and *dmrt1*, leading to testis formation. In *Xenopus*, DM-W antagonizes the male-promoting DMRT1 pathway and promotes ovarian differentiation, including activation of the ovarian factor *cyp19a1*. Solid arrows indicate experimentally demonstrated direct regulatory relationships. Dashed arrows indicate regulatory relationships that are strongly supported and may be direct, but for which direct transcriptional regulation has not yet been conclusively demonstrated. Blunt-ended lines indicate inhibitory or antagonistic effects.

In this review, we examine the evolutionary trajectories of three independently evolved vertebrate MSDGs—*Sry* in mammals, *dmy* in medaka fishes, and *dm-W* in *Xenopus* frogs. Although all three genes function as upstream master regulators of gonadal sex determination, they act through distinct regulatory architectures, as summarized in Figure 1. Despite their distinct molecular origins and evolutionary histories, these genes show striking parallels in their emergence, functional modification, and subsequent relaxation of selective constraint. We refer to this recurrent trajectory as “Jump-Decline” evolution: a newly emerging MSDG undergoes a functional or regulatory “Jump” that enables it to cross the threshold required for sex determination and become established within a population; once established, it may experience relaxed selective constraint, permitting gradual functional “Decline” while sufficient activity is retained to maintain the developmental switch. During this phase, newly emerging MSDG candidates may become established through competition within a population and replace the ancestral determinant while it still retains sufficient sex-determining activity. Such replacement may permit further functional decay or loss of the ancestral MSDG. By comparing these three systems, we explore how threshold-dependent function, relaxed selection, sex-chromosome architecture, and genetic turnover interact to shape the birth, persistence, and replacement of vertebrate master sex-determining genes.

By focusing on these three independently evolved transcription factor-type MSDGs, we do not intend to propose Jump–Decline evolution as a universal trajectory for all genes involved in sex determination. Rather, we ask whether newly evolved transcription factor-type MSDGs that acquire a relatively specialized role as upstream developmental switches share a characteristic evolutionary trajectory after their establishment. Accordingly, transitions between environmental and genetic sex determination are not considered in detail here, because the present review focuses specifically on the molecular evolutionary trajectories of newly evolved transcription factor-type MSDGs after their recruitment into genetic sex-determining systems. The principal contribution of this review is the temporal integration of MSDG establishment, subsequent relaxation of functional constraint, and eventual regulatory turnover into a common evolutionary framework. This framework also generates testable questions concerning the conditions under which functional decline occurs and whether such decline facilitates subsequent MSDG replacement.

## 2. Molecular Evolution of *Sry* in Therian Mammals

The discovery of *Sry* (*sex-determining region of Y*) provided the molecular basis for understanding male sex determination in therian mammals. In 1990, *SRY* was identified within the human Y-chromosomal region associated with testis determination and was shown to encode a protein containing a conserved high-mobility group (HMG) DNA-binding domain [11]. Genetic evidence from human XY individuals with disorders of sex development further supported its identification as the long-sought testis-determining factor (TDF) [12,13]. Direct functional evidence followed in 1991, when Koopman et al. demonstrated that introduction of a mouse *Sry* genomic fragment into chromosomally XX embryos was sufficient to induce testis differentiation and subsequent male development [14]. Together, these studies established *Sry* as the male-determining MSDG in therian mammals.

Subsequent genetic and molecular studies have placed SRY at the apex of the mammalian testis-determining pathway. During a restricted developmental window, *Sry* is transiently expressed in the supporting-cell lineage of the embryonic gonad, where its protein product acts as a transcription factor to initiate testis differentiation, principally through activation of *Sox9* [15,16,17,18,19,20,21,22].

Before overt sex determination, *Sox9* is already expressed at a low level in the bipotential gonads of both sexes. In XY gonads, transient SRY activity, together with other regulatory factors such as SF1, promotes a rapid increase in *Sox9* expression during a critical developmental window [17,19,20,21,22]. Once SOX9 expression reaches a sufficiently high level, positive-feedback and downstream regulatory networks stabilize SOX9 expression and promote Sertoli-cell differentiation and testicular development. Thus, the critical developmental threshold is most directly manifested at the level of effective *Sox9* activation rather than as a simple threshold of SRY biochemical activity itself.

Thus, SRY provides a particularly clear example of a master developmental switch: a transiently expressed transcription factor whose activity initiates a self-reinforcing downstream pathway. This regulatory architecture provides an important context for understanding both the evolutionary origin of *Sry* and subsequent changes in its molecular function. We therefore first consider how *Sry* emerged and became established as the MSDG in the therian ancestor.

### 2.1. Origin and “Jump”: Regulatory Innovation and Establishment

*Sry* is thought to have originated in the common ancestor of therian mammals through evolutionary modification of an ancestral *Sox3*-related allele or locus [12,13,23,24]. Relative to the ancestral *Sox3*-related locus, the emerging *Sry* locus became associated with the male-specific Y-chromosomal lineage and acquired a regulatory context that enabled transient expression in gonadal supporting-cell precursors during the critical period of testis determination. Its initial establishment as an MSDG likely depended on regulatory reorganization around the ancestral locus, potentially involving chromosomal rearrangements that altered its transcriptional context. The acquisition of an appropriate cis-regulatory environment may have enabled spatially and temporally restricted expression during the sex-determining period, thereby recruiting an ancestral SOX3-like factor to the top of the male developmental pathway. The observation that ectopic *Sox3* expression can induce testis development in mice [24] supports the plausibility of this regulatory route to the emergence of *Sry*. This regulatory transition can be regarded as an initial “Jump,” in which a pre-existing transcription factor acquired a novel developmental role through changes in its expression and position within the regulatory network.

**Figure 2:**
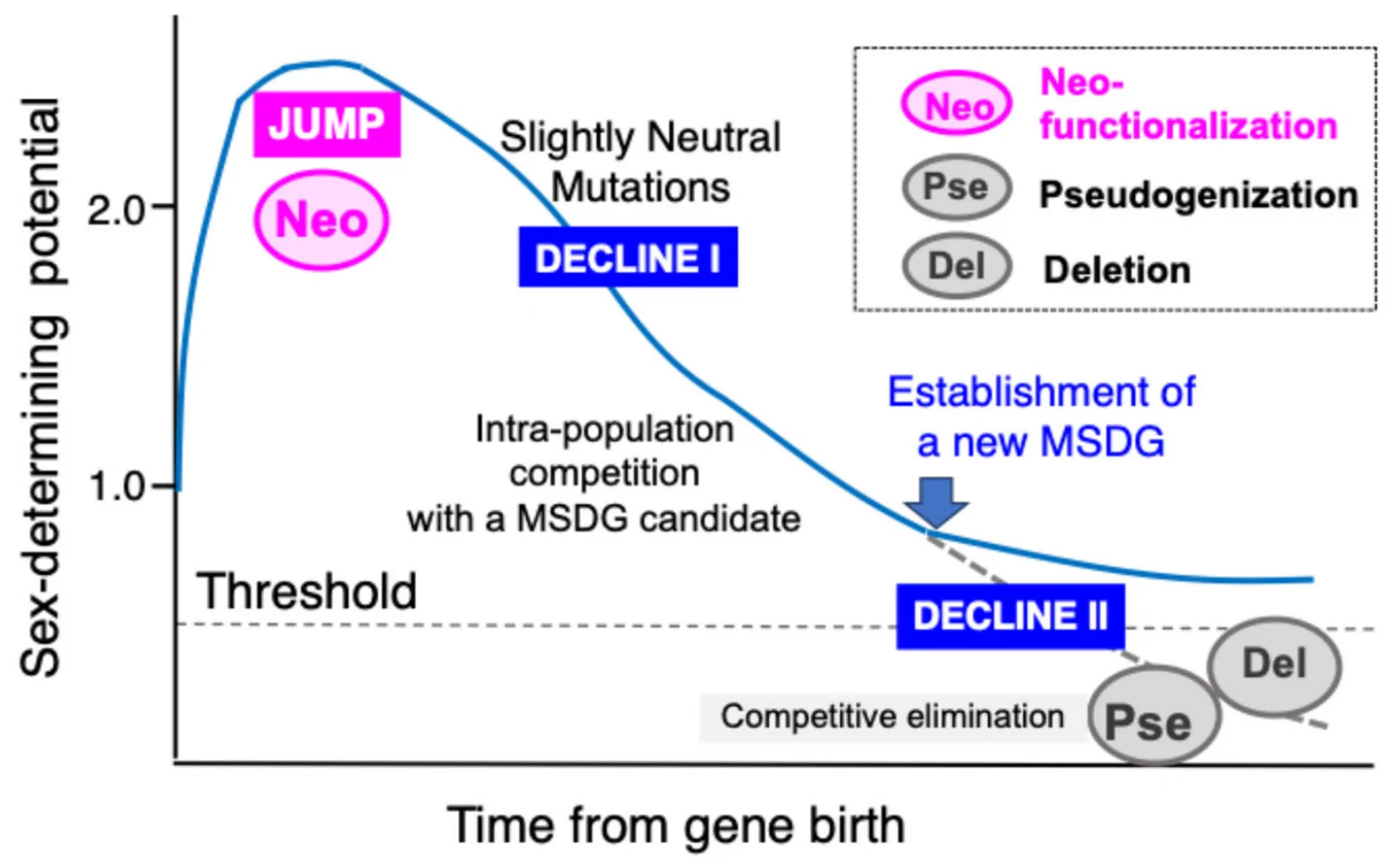
Jump–Decline dynamics in the molecular evolution of MSDGs. Conceptual model illustrating the evolutionary trajectories of master sex-determining genes (MSDGs). Following gene birth, sex-determining activity rapidly increases through neofunctionalization (“Jump”), followed by a gradual “Decline I” phase driven by the accumulation of slightly deleterious or nearly neutral mutations under relaxed purifying selection. During this phase, newly emerging MSDG candidates may become established through competition with the ancestral MSDG within a population, while the ancestral determinant still retains sufficient activity for normal sex determination. Replacement by a new MSDG initiates “Decline II” of the ancestral determinant, in which loss of its master sex-determining role may be followed by further functional decay, pseudogenization, or deletion. This recurrent process of innovation, relaxation, and renewal may underlie repeated turnover of sex-determining genes during vertebrate evolution. The vertical axis represents relative sex-determining activity of the MSDG and should not be interpreted as organismal fitness.

The establishment of *Sry* as the MSDG would also have required the transmission and spread of the new sex-determining system within ancestral populations. Appropriately timed expression and sufficient molecular activity to cross the threshold for male pathway initiation would have provided the functional basis for its establishment and maintenance. From the perspective of “Jump-Decline” evolution, the “Jump” phase therefore encompasses both molecular innovation and population-level establishment of a newly evolved sex-determining switch (Figure 2). Once SRY activity reliably exceeded the functional threshold required to initiate male development, however, further increases in molecular performance may have provided little additional fitness advantage. This transition from innovation to functional sufficiency provides the evolutionary context for the subsequent relaxation of selective constraint and the “Decline” phase discussed below.

### 2.2. Relaxed Constraint and Tolerated Functional Decline of Sry (Decline I)

Following its establishment as an MSDG, *Sry* accumulated substantial sequence variation, including numerous amino acid substitutions, during mammalian evolution [8]. Regions outside the HMG box are highly divergent and often cannot be reliably aligned across distantly related mammalian orders, although recognizable homology is retained among more closely related taxa, such as within primates [8,9]. Focusing on the conserved HMG box region, our recent analyses of eutherian mammals revealed a marked relaxation of purifying selection on *Sry* relative to its ancestral gene *Sox3*, as indicated by a substantially elevated dN/dS ratio [9]. Functional assays further showed that SRY proteins from some species retain DNA-binding activity comparable to that of the reconstructed ancestral form, whereas others exhibit significantly reduced activity. These reductions were particularly evident in afrotherian lineages. Phylogenetic and functional analyses indicated that reductions in activity resulted from multiple amino acid substitutions at different evolutionary stages, supporting repeated lineage-specific functional decline rather than a single ancestral reduction in activity [9].

SRY expression has also been reported outside the embryonic gonadal supporting-cell lineage, including in the adult brain and testis. Although experimental evidence supports functional effects of SRY in the brain in some mammals, the extent to which such extra-gonadal functions are evolutionarily conserved remains unclear. In contrast, SOX3 retains well-established functions in both the nervous system and spermatogonial development, indicating broader pleiotropic constraints on the ancestral factor [25].

We termed this process “tolerated functional decline” [9], whereby mildly deleterious or nearly neutral substitutions can accumulate under relaxed selective constraint while sufficient gene function is retained. Within the “Jump-Decline” framework, this represents “Decline I,” a phase in which molecular activity can progressively decrease while remaining above the functional threshold required to initiate male development. The threshold-dependent nature of *Sry*-mediated sex determination provides a plausible explanation for this evolutionary tolerance. Once SRY activity is sufficient to trigger the downstream testis-determining pathway, further increases in molecular performance may confer little additional fitness benefit, and moderate reductions in activity may be tolerated without compromising sex-determining output. Compared with the broader developmental functions of SOX3, the more restricted regulatory role of SRY may further contribute to this relaxation of selective constraint.

### 2.3. Loss of Sry and Turnover of the Sex-Determining System (Decline II)

Although *Sry* has been retained as the MSDG in the great majority of eutherian mammals, complete loss of *Sry* has been documented only in a small number of exceptional rodent lineages. These include the Japanese spiny rats *Tokudaia osimensis* and *T. tokunoshimensis*, as well as several mole vole species of the genus *Ellobius* [26,27,28]. Although rare, these cases demonstrate that even the long-established mammalian *Sry*-dependent sex-determining system can undergo evolutionary replacement. The long-term retention of *Sry* in most mammals may be due, at least in part, to its integration into a highly differentiated XY chromosome system, which can constrain replacement of an established MSDG [9].

In *T. osimensis*, both the Y chromosome and *Sry* have been completely lost, yet males develop normally through an alternative genetic mechanism [27,29]. Recent genomic analyses identified a male-specific duplication of a cis-regulatory element upstream of *Sox9* [30]. This duplicated element functions as a testis enhancer and is proposed to elevate *Sox9* expression in the absence of SRY, effectively bypassing the ancestral requirement for SRY-mediated activation of *Sox9*. A similar principle has recently been demonstrated experimentally in mice. Subtle sequence changes in the *Sox9* enhancer Enh13, including a single-nucleotide insertion, can elevate *Sox9* expression in the absence of SRY and induce XX male development [31]. These findings illustrate that the critical *Sox9*-activation threshold can be reached through alternative upstream genetic mechanisms without extensive reorganization of the downstream testis-differentiation network. Thus, evolutionary turnover appears to have replaced the upstream trigger while retaining much of the conserved testis-differentiation network. Loss of both the Y chromosome and *Sry* has also occurred in mole voles of the genus *Ellobius* [26,32], although the alternative master sex-determining mechanisms in these species remain unresolved.

Within the Jump–Decline framework, such cases can be regarded as “Decline II,” in which establishment of an alternative sex-determining mechanism renders the ancestral MSDG dispensable, permitting its subsequent loss. Importantly, the available evidence does not demonstrate that *Sry* loss in *Tokudaia* or *Ellobius* was preceded by the measurable functional decline described above as “Decline I.” These cases therefore illustrate the endpoint of turnover without establishing a continuous “Decline I-Decline II” sequence in these lineages. Nevertheless, the independent loss of *Sry* in these rodents illustrates an important feature of MSDG evolution: once an alternative genetic mechanism can reliably activate the downstream sex-determining pathway, even a long-established master regulator can become dispensable and ultimately be lost (Table 1).

## 3. Molecular Evolution of *dmy* in *Oryzias* Fishes

The identification of *dmy* in medaka provided the first molecular identification of an MSDG in a non-mammalian vertebrate and demonstrated that vertebrate sex determination can be controlled by independently evolved upstream regulators. In 2002, through positional cloning of the Y-chromosomal sex-determining region in *Oryzias latipes*, Matsuda et al. identified a Y-specific gene encoding a Doublesex and Mab-3 (DM) domain transcription factor, which they named *dmy* [33]. Naturally occurring XY females with either a disruptive mutation in *dmy* or reduced *dmy* expression provided strong genetic evidence that the gene is required for male development. In parallel, Nanda et al. independently identified the same Y-linked gene as a duplicated copy of *dmrt1*, initially termed *DMRT1Y* and subsequently widely referred to as *dmrt1bY* [34]. Together, these studies established *dmy/dmrt1bY* as the male-determining MSDG of medaka.

Subsequent genetic and developmental analyses have shown that *dmy* is expressed in somatic cells of the developing XY gonad before overt testicular differentiation and acts at the top of the male developmental pathway [33,35]. A loss or reduction in *dmy* function can result in XY female development, whereas appropriate expression of functional *dmy* normally directs testis differentiation [33,36,37]. Unlike *Sry*, which arose in the therian lineage from a *Sox3*-related ancestral locus, *dmy* originated relatively recently through duplication and translocation of an ancestral *dmrt1* gene [34]. Its comparatively recent origin, together with the presence of closely related *Oryzias* species with alternative sex-determining systems, makes *dmy* particularly informative for reconstructing the molecular events underlying the birth, establishment, functional modification, and eventual turnover of an MSDG [38,39,40,41,42,43,44,45,46].

### 3.1. Origin and Establishment (“Jump” Phase)

*Oryzias* fishes possess largely homomorphic sex chromosomes and exhibit repeated turnover of MSDGs within the genus. Functionally validated MSDGs include *dmy* in *O. latipes*, *O. sakaizumii*, and *O. curvinotus*, *gsdfY* in *O. luzonensis*, and *sox3Y* in *O. dancena* [47,48,49,50,51,52,53]. The *dmy* gene originated approximately 10 million years ago through duplication and translocation of the ancestral *dmrt1* gene, generating a Y-linked copy that subsequently acquired a male sex-determining function [33,34,40,46] (Table 1). Compared with the ancestral *dmrt1* locus, *dmy* acquired an earlier developmental expression pattern in gonadal somatic cells, allowing the duplicated copy to function as an upstream male-determining switch [43]. Regulatory innovation appears to have been critical for the establishment of *dmy*. Transposable element insertions, including *Izanagi* and *Rex1*, have been implicated in the formation or modification of cis-regulatory sequences upstream of *dmy*, contributing to an expression pattern appropriate for male determination during the critical developmental window [50,54].

Within the “Jump-Decline” framework, acquisition of this novel regulatory configuration represents a regulatory “Jump” that recruited the duplicated *dmrt1* copy to the top of the male sex-determining pathway. At the protein level, a serine-to-threonine substitution at position 15 (S15T) within the DM domain increases DNA-binding activity and transcriptional activity [10]. This change represents an additional biochemical “Jump” that may have enhanced the ability of the newly evolved DMY protein to reliably activate the male developmental program.

Thus, the establishment of *dmy* appears to have involved multiple layers of evolutionary innovation. Duplication and translocation generated the genomic substrate, regulatory remodeling enabled appropriate expression during sex determination, and protein-level modification enhanced molecular activity. Together, these changes enabled *dmy* to cross the functional threshold required for male determination and become established as the MSDG in the ancestral population (Figure 2). Once this new sex-determining system was established, however, further increases in DMY activity may have provided little additional fitness advantage, creating conditions that permitted the subsequent relaxation of selective constraint and functional “Decline”.

### 3.2. Molecular Divergence and Possible Tolerated Functional Decline (Decline I Phase)

Across *O. curvinotus*, *O. latipes*, and *O. sakaizumii*, *dmy* exhibits accelerated sequence evolution relative to its ancestral gene *dmrt1*, including multiple amino acid substitutions within the conserved DM domain [8,10]. Among these substitutions, S15T represents a function-enhancing change associated with the initial biochemical “Jump.” This substitution is particularly notable because it also occurred independently in the female-determining gene *dm-W*. In contrast, many subsequent amino acid substitutions show no evidence of comparable functional enhancement, a pattern consistent with evolution under relaxed selective constraint.

Within the “Jump-Decline” framework, the accelerated sequence evolution of *dmy* and the experimentally demonstrated effects of amino acid substitutions are compatible with a potential “Decline I” phase, in which sequence changes may accumulate under relaxed selective constraint while sufficient sex-determining activity is retained [8,10]. Because DMY functions as an upstream developmental switch, moderate reductions in molecular activity could in principle be tolerated as long as the downstream male pathway continues to be reliably activated. However, direct published evidence for a progressive, stepwise reduction in DMY activity over evolutionary time is not yet available. Thus, “Decline I” in DMY should currently be regarded as an evolutionary interpretation consistent with the available evidence rather than as a fully demonstrated functional trajectory.

### 3.3. Replacement by gsdfY and Loss of dmy (“Decline II” Phase)

In the lineage leading to *O. luzonensis*, the emergence and establishment of *gsdfY* are thought to have led to the functional replacement of *dmy* as the MSDG [54] (Table 1). No identifiable *dmy* sequence has been reported in this species, but the precise molecular route by which the ancestral gene disappeared remains unresolved; pseudogenization followed by extensive sequence degeneration and physical genomic deletion are both possible. Within the “Jump-Decline” framework, this transition represents “Decline II,” in which an established MSDG becomes dispensable after an alternative determinant assumes control of the same developmental pathway. Once *gsdfY* acquired sufficient sex-determining activity and became established as the new male-determining switch, selective pressure maintaining the sex-determining function of *dmy* would have been relaxed, allowing its subsequent loss.

Within this model, the transition can be conceptualized as population-level competition between alternative sex-determining genes within a population, leading to a shift in regulatory dominance within the sex-determining network. Such competition does not require direct biochemical antagonism between the encoded proteins. A newly evolved determinant can assume control of the male developmental pathway while the ancestral MSDG still retains sufficient activity and much of the downstream regulatory architecture remains conserved. In this sense, the replacement and subsequent loss of *dmy* represent turnover of the master regulatory position itself—a genetic takeover in which one upstream sex-determining switch is replaced by another (Figure 2).

Several features of the *dmy* system may facilitate such turnover. These include its operation within a threshold-dependent developmental network, in which changes in the expression or molecular activity of a candidate determinant may enable it to cross the threshold required for male determination, and the presence of largely homomorphic sex chromosomes in *Oryzias*. Such chromosomal architecture may impose fewer constraints on the establishment of alternative MSDGs than highly differentiated sex chromosomes. Together, these features may contribute to the repeated turnover of sex-determining genes observed within the genus.

## 4. Molecular Evolution of *dm-W* in Allotetraploid *Xenopus* Frogs

The discovery of *dm-W* marked the first molecular identification of a female-determining MSDG in amphibians and revealed a distinct mechanism of vertebrate sex determination involving a W-linked regulator. In 2008, we isolated *dm-W* from *Xenopus laevis* as a female-specific, W-linked gene encoding a protein with a Doublesex and Mab-3 (DM) DNA-binding domain [55]. *dm-W* is transiently expressed in the primordial gonads of ZW tadpoles during the critical period of sex determination, whereas its paralog *dmrt1* is expressed in the gonads of both sexes, with expression maintained during subsequent testicular development. Functional analyses using transgenic tadpoles demonstrated that ectopic expression of *dm-W* in genetically male ZZ individuals induced ovarian differentiation, including the formation of ovarian cavities and primary oocytes, providing direct evidence that DM-W promotes female development [55].

Subsequent molecular and functional analyses further established *dm-W* as a female MSDG. DM-W and DMRT1 have closely related DM domains and recognize overlapping DNA sequences, but DM-W lacks the C-terminal transactivation region characteristic of DMRT1. Functional experiments suggested that DM-W functionally antagonizes the male-promoting activity of DMRT1, thereby shifting the developmental balance toward ovarian differentiation. Early loss-of-function studies using *dm-W* knockdown in ZW individuals provided evidence that the gene is required for normal female development and primary ovary formation [56]. More recently, CRISPR/Cas9-mediated disruption of *dm-W* resulted in complete female-to-male sex reversal in genetically ZW individuals, yielding fertile phenotypic males and providing strong genetic confirmation of its essential role in female sex determination [57]. Thus, whereas *Sry* and *dmy* act as male-determining MSDGs, *dm-W* functions as a dominant female-determining switch whose protein product promotes ovarian development by counteracting the conserved male-promoting activity of DMRT1.

The distinctive molecular structure of DM-W, the W-linked inheritance of *dm-W*, and the antagonistic relationship between DM-W and DMRT1 make this system particularly informative for understanding how gene duplication and molecular reorganization can generate a new MSDG capable of assuming control of an existing sex-determining network.

### 4.1. Origin and Establishment (“Jump” Phase)

Amphibians generally exhibit genetic sex determination with largely homomorphic sex chromosomes, and turnover of sex-determining systems is thought to be frequent, although relatively few cases have been characterized at the molecular level. We identified *dm-W* as a W-specific, female-determining MSDG in *Xenopus* [55,56,58]. The allotetraploid *Xenopus* lineage is estimated to have originated approximately 17–18 million years ago through interspecific hybridization, bringing together the ancestral genomes that gave rise to the L and S subgenomes [59]. Subsequent genomic reorganization and transposable element activity within the hybrid genome may have contributed to the emergence of *dm-W* on chromosome 2L [60,61,62].

The origin of *dm-W* involved the assembly of sequence components with distinct evolutionary origins. Exons 2–3, which include the region encoding the DM DNA-binding domain, were derived from a partial duplication of *dmrt1.S* in the S subgenome, whereas the noncoding exon 1 and its associated regulatory region were newly acquired during the emergence of *dm-W* [61,63]. This novel regulatory architecture appears to have enabled the highly restricted expression pattern characteristic of *dm-W*: transient expression in gonadal somatic cells during the critical period of sex determination. Within the “Jump-Decline” framework, acquisition of exon 1 and its associated cis-regulatory environment therefore represents a regulatory “Jump” that recruited the partially duplicated *dmrt1* copy into the female sex-determining pathway.

A further structural and functional innovation involved exon 4, which originated from the DNA transposon *hAT10* and was subsequently co-opted as a protein-coding sequence [61]. Functional analyses demonstrated that the *hAT10*-derived C-terminal region enhances the DNA-binding activity of DM-W in vitro, indicating that transposon recruitment contributed directly not only to the structural formation of the gene but also to enhancement of its molecular function. Thus, *dm-W* differs fundamentally from its ancestral *dmrt1.S* locus: it retained only part of the ancestral coding region, did not retain the ancestral promoter configuration, acquired a novel exon 1 and TATA-type promoter, and incorporated an *hAT10*-derived sequence as a new protein-coding exon [61,63]. Recent knockout analyses further revealed functional divergence between the two *dmrt1* homeologs in *X. laevis*: *dmrt1.S*, from which *dm-W* was partially derived, is dispensable for fertility in both sexes, whereas *dmrt1.L* has acquired an essential role in male fertility [64]. In addition, a serine-to-threonine substitution at position 15 (S15T) within the DM domain increases DNA-binding activity [10]. Remarkably, the same amino acid change independently occurred in the male-determining MSDG DMY in *Oryzias*, representing parallel biochemical innovation in two independently evolved *dmrt1*-derived MSDGs (Table 1).

Together, these events indicate that the establishment of *dm-W* involved multiple layers of evolutionary innovation. Partial duplication of *dmrt1.S* provided the ancestral DNA-binding module; acquisition of exon 1 and its associated cis-regulatory architecture enabled expression in gonadal somatic cells during the sex-determining period; incorporation of an *hAT10*-derived coding exon enhanced DNA-binding activity; and the S15T substitution further increased this activity. These structural, regulatory, and biochemical innovations collectively provided the functional basis for *dm-W* to cross the threshold required for female determination and become established as the MSDG in the allotetraploid lineage (Figure 2). Thus, *dm-W* illustrates how genome reorganization following interspecific hybridization and polyploidization, together with transposable element recruitment, can contribute to the emergence of a novel master regulator of vertebrate sex determination.

### 4.2. Lineage-Specific Relaxation, Replacement, and Loss of dm-W (Decline I–II)

In *X. laevis*, *dm-W* shows substantially greater protein-coding sequence divergence than its ancestral gene *dmrt1*, a pattern consistent with relaxation of selective constraint following its establishment as a female-determining MSDG. Among the substitutions examined experimentally, S15T represents a function-enhancing change associated with the initial biochemical “Jump,” whereas most other substitutions show no evidence of comparable functional enhancement [10]. These patterns are compatible with “Decline I,” in which sequence changes accumulate under relaxed selective constraint while the sex-determining function of *dm-W* is retained. However, direct evidence for a progressive reduction in DM-W molecular activity remains limited. Thus, “Decline I” in *dm-W* should currently be regarded as a plausible evolutionary interpretation rather than an established functional trajectory. Further ancestral sequence reconstruction and functional analyses are needed to determine whether DM-W underwent a measurable, stepwise reduction in molecular activity following its initial biochemical “Jump”.

Evidence for turnover of the *dm-W*-dependent sex-determining system is stronger. Comparative analyses across allotetraploid *Xenopus* species have revealed extensive lineage-specific variation in the distribution of *dm-W*: the gene is retained as a female-specific sequence in only a subset of species, occurs in both sexes in others, and is absent from several lineages [57] (Table 1). These patterns indicate that the association between *dm-W* and female determination has not been consistently maintained during the diversification of allotetraploid *Xenopus*. Variation in the distribution and female specificity of *dm-W* occurs alongside extensive sex-chromosome turnover across the genus. An analysis of 929 individuals from 19 *Xenopus* species identified at least eight largely nonhomologous sex-associated genomic regions, with evidence for multiple changes in sex-chromosome identity and two independent origins of male heterogamety [65]. Together, variation in the female specificity of *dm-W* and the diversity of sex-associated regions argue against a simple model in which *dm-W* repeatedly relocates to new sex chromosomes. Instead, the available evidence supports repeated replacement of sex-determining systems during *Xenopus* diversification [65,66,67,68]. Additional studies further illustrate the diversity of sex-determining systems within *Xenopus*. In experimental hybrids between *X. laevis* and *X. tropicalis*, DM-W can dominate over the male-determining sex chromosomes of *X. tropicalis*, demonstrating functional interactions between independently evolved sex-determining systems [69]. Moreover, recent analyses of species in the subgenus *Silurana* revealed that an unusual triple sex-chromosome system can persist through allotetraploidization, indicating that sex-chromosome evolution in *Xenopus* includes not only recurrent turnover but also lineage-specific persistence of complex systems [70]. Together, these findings emphasize that the evolutionary fate of *dm-W* should be interpreted within the broader diversity and dynamic history of sex-determining systems across the genus.

Within the “Jump-Decline” framework, these lineage-specific changes are consistent with “Decline II,” in which an alternative sex-determining mechanism renders the ancestral MSDG dispensable, allowing loss of its master regulatory role, with or without subsequent loss of the gene itself (Figure 2). The occurrence of *dm-W* in both sexes, or its complete absence, together with the presence of alternative sex-associated regions, suggests that other genetic mechanisms have assumed control of primary sex determination in these lineages. Although loss of female specificity alone does not establish the complete loss of DM-W function, it indicates that the ancestral W-linked, female-specific configuration is no longer maintained. As in the loss of *Sry* in some rodents and replacement of *dmy* by *gsdfY* in *Oryzias*, the lineage-specific loss or loss of female specificity of *dm-W* is consistent with turnover of the upstream regulatory trigger while the downstream developmental network remains capable of directing sexual differentiation. Thus, although a continuous “Decline I-Decline II” trajectory has yet to be demonstrated for *dm-W*, comparative evidence supports the view that an established female-determining MSDG can lose its master regulatory role and ultimately be replaced or lost during vertebrate evolution.

## 5. Integrative Summary: Shared “Jump-Decline” Dynamics of Vertebrate MSDGs

Comparative analyses of *Sry*, *dmy*, and *dm-W* reveal parallels in the evolutionary histories of three independently evolved vertebrate MSDGs (Table 1). In each case, molecular innovations involving gene regulation, protein function, gene structure, or combinations of these processes contributed to the emergence and establishment of a new master determinant. Within the “Jump-Decline” framework, innovations that enabled a candidate determinant to cross the functional threshold required to initiate female or male development, together with its establishment within a population, constitute the “Jump” phase.

Following establishment, the three MSDGs show different levels of evidence for relaxed selective constraint and functional decline. For SRY, comparative and functional analyses provide direct evidence for lineage-specific reductions in molecular activity under relaxed selective constraint [9]. For DMY and DM-W, molecular evolutionary analyses revealed accelerated amino acid substitution relative to their ancestral DMRT1-related genes, including substitutions within the conserved DNA-binding DM domain [8]. Notably, the parallel S15T substitution that occurred early in the evolution of both DMY and DM-W was positively selected and experimentally shown to enhance molecular activity [10], consistent with an initial biochemical “Jump.” Numerous additional amino acid substitutions subsequently accumulated in both proteins, but their functional consequences remain largely unresolved. These patterns are compatible with subsequent relaxation of functional constraint and possible tolerated functional decline, although direct evidence for progressive decline is currently available only for SRY. Thus, “Decline I” is experimentally demonstrated for SRY, whereas for DMY and DM-W it remains an evolutionary hypothesis supported by patterns of molecular evolution but requiring direct functional testing. Accordingly, we regard Jump–Decline evolution as a testable conceptual framework supported to different degrees by these representative cases, rather than as an established general trajectory of vertebrate MSDGs. A further shared feature is that even an established MSDG can become evolutionarily dispensable. *Sry* has been lost in several rodent lineages, *dmy* is thought to have been replaced by *gsdfY* and subsequently lost in the lineage leading to *O. luzonensis*, and *dm-W* has lost its female-specific association or disappeared in multiple allotetraploid *Xenopus* lineages. These patterns support turnover of the ancestral sex-determining system, although loss of female specificity alone does not establish complete loss of DM-W function. Within the “Jump-Decline” framework, such turnover is consistent with “Decline II,” in which an alternative determinant or regulatory mechanism assumes control of sex determination, rendering the ancestral MSDG dispensable and permitting its subsequent functional decay or loss.

Taken together, these systems support a model in which functional or regulatory innovation enables MSDG establishment, after which relaxed selective constraint may permit tolerated functional decline; in some lineages, an alternative sex-determining mechanism subsequently replaces the ancestral master determinant (Figure 2). The threshold-dependent architecture of sex determination provides a plausible mechanistic basis for tolerated decline: once an MSDG reliably initiates the downstream developmental pathway, further increases in molecular activity may confer little additional fitness benefit, while moderate reductions may be tolerated without compromising sex-determining output. Replacement need not await a decline below the functional threshold; an alternative determinant may become established while the ancestral MSDG retains sufficient activity. Whether turnover occurs is therefore likely to depend on additional factors, including the emergence and spread of alternative determinants and the chromosomal context of the ancestral MSDG. Although a continuous “Jump-Decline I-Decline II” trajectory has not been demonstrated in every system, these comparative patterns provide a foundation for considering the broader evolutionary implications of the model in the Discussion below.

## 6. Discussion

### 6.1. Why Do MSDGs Undergo Functional “Jump”?—Threshold Crossing During the Establishment of a New Master Regulator

The emergence of a new MSDG requires more than molecular novelty: the candidate determinant must exert sufficient regulatory influence to direct an existing developmental network toward male or female development. The evolutionary histories of *Sry*, *dmy*, and *dm-W* suggest that this threshold can be crossed through different combinations of regulatory, structural, and biochemical changes. Thus, the “Jump” is best understood as a functional transition in which a candidate determinant acquires sufficient activity, at the appropriate developmental time and place, to initiate the male or female pathway. This transition provides the functional basis for its establishment as a new master regulator.

The relevant threshold is likely to depend on the properties of the developmental network and the timing, location, and level of MSDG expression, as well as the biochemical activity of its protein product. In mice, insufficient *Sry* expression during the restricted sex-determining window can prevent testis determination, providing experimental support for the importance of appropriately timed expression in reaching the threshold required to initiate the male developmental pathway [71]. At the downstream network level, the functional consequence of this SRY-dependent threshold is manifested in whether *Sox9* expression is elevated to a sufficient level to initiate and stabilize the testis-determining pathway [19,20,21,22]. SRY acts transiently upstream to elevate *Sox9* expression during the critical developmental window [17,19,20,21,22], and once a sufficient level of *Sox9* expression is reached, downstream and positive-feedback mechanisms stabilize the testis-determining pathway [19,20,22]. Thus, reductions in SRY molecular activity may be tolerated as long as sufficient *Sox9* expression is still achieved [9]. In contrast, SOX9 itself has broad pleiotropic developmental functions across multiple organs [72] and is therefore expected to experience stronger constraint on protein function than the more specialized upstream SRY trigger.

### 6.2. Why Can MSDGs Decline?—Functional Sufficiency and Relaxed Selection

A central question raised by the evolutionary trajectories of *Sry*, *dmy*, and *dm-W* is why genes with such critical developmental roles can tolerate substantial molecular change. A plausible explanation lies in the threshold-dependent nature of MSDG-mediated sex determination. Once an MSDG provides sufficient activity to initiate the downstream male or female developmental pathway, further increases in molecular activity may confer little additional fitness benefit. Selection may therefore act primarily to preserve reliable activation of the developmental pathway rather than to maximize biochemical activity. Under this regime, mutations that moderately reduce molecular activity may have little or no effect on fitness, allowing nearly neutral or mildly deleterious variants to accumulate through genetic drift under relaxed purifying selection [73,74]. Importantly, molecular activity and fitness are not equivalent variables in this framework. The vertical axis in Figure 2 represents the relative sex-determining activity of an MSDG, not organismal fitness. As long as MSDG activity remains above the developmental threshold required for reliable sex determination, even a substantial reduction in molecular activity may have little or no phenotypic consequence and therefore little or no effect on individual fitness. Fitness consequences would be expected to become more pronounced only when the activity of the upstream determinant approaches a level at which reliable initiation of the downstream pathway is compromised. This process provides a plausible explanation for the tolerated functional decline demonstrated for SRY and hypothesized for DMY and DM-W.

An important boundary condition for such tolerated functional decline is the degree of pleiotropic constraint acting on the transcription factor. Ancestral transcription factors commonly regulate numerous downstream genes and function in multiple developmental or cellular contexts. Their DNA-binding domains are therefore generally subject to strong purifying selection because amino acid substitutions that alter DNA recognition or binding activity can affect multiple target genes and biological processes.

Conserved developmental regulators such as DMRT1 and FOXL2 illustrate this contrast. In particular, the ancestral *dmrt1* gene retains functions beyond the initial determination of gonadal fate, including roles in gonadal development and maintenance and in germ-cell development in vertebrates [35,50,57]. Such pleiotropic functions are expected to maintain strong purifying selection on DMRT1 and constrain functional divergence. FOXL2 likewise retains multiple developmental functions beyond the initial regulation of gonadal fate, further illustrating how pleiotropic constraints can contribute to long-term functional conservation.

By contrast, a newly evolved MSDG may become specialized for a relatively restricted developmental role, acting during a limited developmental window to activate a small number of critical downstream targets sufficient to initiate the male or female pathway. Such specialization may reduce some of the pleiotropic constraints that act on its ancestral transcription factor. Consequently, even amino acid changes within the DNA-binding domain may be tolerated to a greater extent, provided that sufficient activity is retained to trigger the downstream sex-determining pathway. We therefore propose that reduced pleiotropic constraint, together with threshold-dependent functional sufficiency, represents an important condition under which tolerated functional decline can occur in newly evolved transcription factor-type MSDGs.

This interpretation links MSDG evolution to relaxed purifying selection and nearly neutral processes. “Decline” does not imply immediate loss of sex-determining function: an MSDG may undergo reductions in molecular activity while remaining fully competent as a developmental switch. Thus, functional decline in this framework refers primarily to a reduction in molecular activity and does not necessarily imply a reduction in organismal fitness or sex-determining efficacy as long as activity remains above the functional threshold. The extent of tolerated decline is likely to depend on the margin by which its effective activity exceeds the sex-determining threshold, as well as on the sensitivity and robustness of the downstream regulatory network [75]. Such a margin may arise during the preceding “Jump” or through subsequent changes in gene expression or network properties. Thus, the threshold-dependent architecture of sex determination may provide a functional buffer that allows molecular activity to decline while reliable sex-determining output is preserved.

### 6.3. From Decline I to Decline II: Replacement, Loss, and Genetic Takeover of MSDGs

“Decline I” does not necessarily lead to replacement or loss of an MSDG. As long as sufficient activity is retained to initiate sex determination reliably, an MSDG with reduced molecular activity can remain stably maintained over extended evolutionary periods. Within the “Jump-Decline” framework, transition to “Decline II” involves the emergence and establishment of an alternative determinant or regulatory mechanism capable of assuming control of sex determination. Once this alternative mechanism becomes established, selection maintaining the sex-determining function of the ancestral MSDG may be further relaxed, permitting additional functional decay or eventual gene loss. The ancestral gene may also persist after losing its master regulatory role. Thus, “Decline II” encompasses turnover of the master regulatory position and the subsequent evolutionary fate of the displaced MSDG.

A different situation may arise when the molecular activity of the ancestral MSDG declines toward the developmental threshold. In this case, the downstream sex-differentiation network itself may remain fully functional, while the upstream trigger provided by the ancestral MSDG becomes less reliable. Stochastic variation in MSDG expression or environmental effects could then increase the probability that the developmental threshold is not consistently crossed, potentially resulting in sex reversal or biased sex ratios. Under such conditions, a newly arisen MSDG with high penetrance that reliably activates the existing downstream pathway and produces a more stable, approximately balanced sex ratio could acquire a selective advantage. Thus, functional decline of the ancestral MSDG may increase the opportunity for establishment of an alternative MSDG even before the ancestral determinant has completely lost its sex-determining function.

Evidence consistent with such turnover comes from the loss of *Sry* in *Tokudaia* and *Ellobius*, the proposed replacement of *dmy* by *gsdfY* in *Oryzias*, and lineage-specific changes in the female specificity and retention of *dm-W* in allotetraploid *Xenopus*. These transitions can be interpreted as genetic takeover, a concept related to our earlier model in which a newly emerging sex-determining gene replaces the ancestral determinant, particularly on poorly differentiated sex chromosomes [8]. In this model, competition between alternative determinants within a population can lead to replacement of the master regulator, potentially accompanied by a change in sex-chromosome identity. Such competition does not require direct biochemical antagonism between their protein products. The “Jump” of a new determinant and “Decline II” of the ancestral MSDG can therefore be viewed as coupled aspects of turnover: establishment of the new switch renders the ancestral determinant dispensable and may permit its subsequent decay or loss (Figure 2). Prior functional decline of the ancestral MSDG may therefore facilitate replacement by reducing the reliability of the upstream sex-determining trigger, but it is not a prerequisite for turnover. An alternative MSDG may also become established while the ancestral determinant remains fully functional, through population-genetic processes that do not require a large selective advantage. A continuous “Decline I-Decline II” sequence should therefore not be assumed for every lineage. Recurrent MSDG turnover may instead reflect varying contributions of functional decline, regulatory innovation, sex-ratio selection, genetic drift, population structure, and changes in selective constraint.

### 6.4. Sex-Chromosome Architecture May Constrain MSDG Turnover

Functional decline of an MSDG does not necessarily lead to its replacement. Whether an established master regulator can be displaced by a newly emerging determinant is likely to depend, in part, on its chromosomal context. We previously proposed that replacement of sex-determining genes occurs more readily in lineages with homomorphic sex chromosomes, whereas progressive sex-chromosome differentiation tends to stabilize the existing system and constrain subsequent replacement [8]. Consistent with this hypothesis, *Oryzias* fishes and allotetraploid *Xenopus*, which generally possess poorly differentiated sex chromosomes, exhibit turnover of sex-determining systems, including replacement or lineage-specific loss of *dmy* and *dm-W*. By comparison, *Sry* has been retained in most therian mammalian lineages within a highly differentiated XY chromosome system, despite substantial molecular divergence and lineage-specific functional decline.

One possible explanation is that progressive recombination suppression and sex-chromosome differentiation integrate the ancestral MSDG into an increasingly specialized genomic context. Degeneration of the Y or W chromosome, divergence in sex-linked gene content, and the evolution of dosage-regulatory mechanisms may increase the genomic consequences of replacing the ancestral sex-determining system [76,77]. Establishment of a new master determinant may therefore depend not only on its ability to initiate the appropriate developmental pathway, but also on its compatibility with existing sex-linked gene content and regulatory relationships. The strength of these constraints is likely to vary with the type of transition and the fate of the ancestral sex chromosomes. Homomorphic sex chromosomes may impose fewer such constraints, allowing alternative MSDGs to become established more readily.

Thus, functional decline and evolutionary turnover should be regarded as distinct but interacting processes. The former may occur under relaxed selective constraint on the MSDG itself, whereas the latter also depends on the emergence and establishment of an alternative determinant within a broader genomic and chromosomal context. Sex-chromosome architecture may therefore influence whether an MSDG undergoing “Decline I” remains the master regulator over extended evolutionary periods or is displaced through genetic takeover. Such replacement need not require prior functional decline but may initiate “Decline II” by rendering the ancestral MSDG dispensable.

### 6.5. Genomic Routes to the Birth of New MSDGs: Duplication, Regulatory Rewiring, and Genome Reorganization

The three MSDGs examined here illustrate distinct genomic routes to the emergence of a new master determinant. *Sry* likely arose through evolutionary modification of an ancestral *Sox3*-related locus, with regulatory reorganization contributing to its recruitment into sex determination. By comparison, *dmy* originated through duplication and translocation of *dmrt1*, followed by regulatory and protein-level changes that enabled its male-determining function. The origin of *dm-W* involved the assembly of components with distinct evolutionary origins, including a partial duplicate of *dmrt1.S*, a newly acquired regulatory region, and a transposon-derived coding exon, in the genomic context of interspecific hybridization and allotetraploidization. Together, these cases suggest that diverse forms of genomic change can generate a candidate regulator capable of crossing the functional threshold required to initiate male or female development.

Gene duplication and genome reorganization may therefore provide important substrates for MSDG innovation. Duplication can reduce constraints on one gene copy while another retains the ancestral function, creating opportunities for functional divergence [1,5,6]. Chromosomal rearrangements can alter the regulatory context of a gene, while transposable elements can contribute both cis-regulatory sequences and novel coding material. Hybridization and polyploidization may further expand opportunities for regulatory and structural innovation by bringing divergent genomes, gene copies, and regulatory systems into the same nucleus. The relative contributions of these processes to the emergence and turnover of vertebrate MSDGs remain to be established. Nevertheless, they provide multiple routes to the functional or regulatory innovations that enable a candidate determinant to cross the sex-determining threshold and, upon establishment within a population, complete the “Jump” phase.

### 6.6. Why Are Master Sex-Determining Genes (MSDGs) So Diverse?

The remarkable diversity of vertebrate MSDGs may reflect an important property of sex-determining networks: relatively conserved downstream developmental pathways can be controlled by evolutionarily replaceable upstream switches. A candidate determinant can acquire control of an existing male or female developmental pathway through appropriate spatiotemporal expression and sufficient regulatory activity, without requiring the entire differentiation program to evolve anew. Gene duplication, regulatory rewiring, chromosomal rearrangement, transposable element recruitment, and other forms of genome reorganization can provide multiple routes to this functional transition.

Once established, an MSDG may experience relaxed selective constraint if moderate reductions in molecular activity do not compromise reliable sex determination, permitting tolerated functional decline (Figure 2). Repeated opportunities for functional or regulatory “Jumps” may enable new determinants to become established and replace ancestral MSDGs, whether or not those ancestral regulators have already undergone measurable functional decline. Replacement may then further relax selection on the sex-determining function of the displaced MSDG, permitting additional decay or loss. The likelihood of turnover is also influenced by genomic context, particularly the degree of sex-chromosome differentiation and the compatibility of a new determinant with existing sex-linked genomic features. Thus, the diversity of vertebrate MSDGs may reflect a fundamental asymmetry in sex-determining networks: relative developmental conservation downstream and evolutionary flexibility upstream.

### 6.7. Evolutionary Implications and Future Perspectives

The “Jump-Decline” dynamics proposed here may extend beyond MSDGs. Although the present framework is developed primarily for newly evolved transcription factor-type MSDGs, similar dynamics may occur in other duplicated or newly recruited regulatory genes under comparable functional conditions. Similar trajectories may occur in genes that acquire novel functions, operate within threshold-dependent regulatory systems, and retain activity above the level required for reliable biological output. Under these conditions, an initial functional or regulatory “Jump” may be followed by relaxed selective constraint, allowing moderate reductions in molecular activity to be tolerated. The relevant threshold may vary among lineages and developmental contexts such that the same gene remains strongly constrained in one lineage but undergoes functional decline, loss, or further innovation in another. Duplicated regulatory genes such as *sox15*, which show lineage-specific variation in retention, pseudogenization, loss, and functional diversification, may provide useful systems for testing whether these outcomes reflect “Jump-Decline” dynamics [78,79].

As a conceptual framework, Jump–Decline evolution generates several testable predictions and comparative questions. Several questions remain central to future studies. First, it will be important to determine whether other independently evolved MSDGs, including *gsdfY*, *sox3Y*, *sdY*, and *amh/amhr2*-derived determinants, show evidence of initial regulatory or biochemical innovation followed by tolerated functional decline. Second, ancestral sequence reconstruction combined with quantitative functional assays can help reconstruct changes in molecular activity over evolutionary time. Establishing how these changes relate to sex-determining thresholds will additionally require experiments that vary gene expression or protein activity in vivo and measure the resulting developmental outcomes. Together, these approaches could define the extent of functional decline that can be tolerated without compromising sex determination. Finally, the relationship between “Decline I” and “Decline II” remains poorly understood. Comparative and population-genetic analyses are needed to determine whether prior functional decline facilitates replacement, or whether turnover commonly occurs while the ancestral MSDG retains substantial activity. Sex-chromosome differentiation, effective population size, genetic drift, hybridization, and genome reorganization may influence both the persistence of an ancestral MSDG and the establishment of an alternative determinant. Expanding these analyses across vertebrate lineages and other regulatory gene families will help establish the scope and limits of “Jump-Decline” evolution as a framework linking functional innovation, relaxed selective constraint, and regulatory turnover.

## Figures and Tables

**Table 1:** Molecular evolution of master sex-determining genes (MSDGs) under the “Jump-Decline” model.

MSDG	Ancestral Origin	Birth	Jump	Sex Chromosome Context	Decline I	Decline II/ Turnover
*Sry*	*Sox3*-related locus	Ancestor of therian mammals	Regulatory innovation	Heteromorphic XY	Direct functional evidence for lineage-specific decline	Rare lineage-specific loss/replacement; continuity from Decline I not demonstrated
*dmy*	Duplicated *dmrt1*	*Oryzias* ancestor	Novel cis-regulation + functional enhancement (aa substitution)	Homomorphic XY	Molecular divergence consistent with relaxed constraint; progressive functional decline not directly demonstrated	Proposed replacement by *gsdfY* in *O. luzonensis*
*dm-W*	Partially duplicated *dmrt1.S* + *hAT10*-derived DNA transposon	Allotetraploid *Xenopus* ancestor	Novel cis-regulation + TE-mediated neofunctionalization + functional enhancement (aa substitution)	Homomorphic ZW	Molecular divergence consistent with relaxed constraint; progressive functional decline not directly demonstrated	Loss of female specificity or gene loss in multiple lineages; turnover inferred

Summary of the molecular features and evolutionary trajectories of representative MSDGs considered within the “Jump-Decline” framework. Each MSDG originated from an ancestral Sox- or Dmrt-type gene through gene duplication, partial duplication, or modification of an ancestral locus, followed by regulatory, structural, and/or biochemical innovation associated with its establishment as a master sex-determining gene. The status of “Decline I” differs among the three systems: lineage-specific functional decline has been directly demonstrated for SRY, whereas in DMY and DM-W molecular divergence is consistent with relaxed selective constraint, but progressive functional decline has not been directly demonstrated. “Decline II” refers to replacement or loss of the master sex-determining role and does not require a preceding demonstrated “Decline I” phase. Abbreviations: aa, amino acid; MSDG(s), master sex-determining gene(s); TE, transposable element.

## Data Availability

No new data were created or analyzed in this study. Data sharing is not applicable to this article.

## Abbreviations

MSDG(s), master sex-determining gene(s); HMG, high-mobility group; DM, a Doublesex and Mab-3; dN/dS, ratio of nonsynonymous/synonymous substitution.
